# miRNA-193a-5p repression of p73 controls Cisplatin chemoresistance in primary bone tumors

**DOI:** 10.18632/oncotarget.10950

**Published:** 2016-07-29

**Authors:** Camille Jacques, Lidia Rodriguez Calleja, Marc Baud'huin, Thibaut Quillard, Dominique Heymann, François Lamoureux, Benjamin Ory

**Affiliations:** ^1^ INSERM, UMR 957, Équipe Labellisée Ligue 2012, Nantes, France; ^2^ Physiopathologie de la Résorption Osseuse et Thérapie des Tumeurs Osseuses Primitives, Université de Nantes, Nantes Atlantique Universités, Nantes, France; ^3^ Nantes University Hospital, Nantes, France

**Keywords:** chemoresistance, microRNAs, p73

## Abstract

Osteosarcoma and Ewing Sarcoma are the two most common types of Bone Sarcomas, principally localized at the long bones of the extremities and mainly affecting adolescents and young adults. Cisplatin is one of the current options in the therapeutic arsenal of drugs available to cure these aggressive cancers. Unfortunately, chemoresistance against this agent is still a major cause of patient relapse. Thus, a better understanding of the molecular pathways by which these drugs induce cancer cell death, together with a better delineation of the origins of chemoresistance are required to improve the success rate of current treatments. Furthermore, as p53 is frequently mutated in Bone Sarcomas, other pathways in these cancers must mediate drug-induced cell death. Here, we demonstrate for the first time that TAp73β, a p53-family protein, is implicated in Cisplatin-induced apoptosis of Bone Sarcomas'. Furthermore, while acquired resistance developed by cancer cells against such drugs can have multiple origins, it is now well accepted that epigenetic mechanisms involving microRNAs (miRNAs) are one of them. We show that miRNA-193a-5p modulates the viability, the clonogenic capacity and the Cisplatin-induced apoptosis of the Bone Sarcoma cells through inhibition of TAp73β. Collectively, these results shed light on the involvement of miR-193a-5p in Cisplatin chemoresistance of Bone Sarcomas', and they open the road to new therapeutic opportunities provided by targeting the miR-193a-5p/TAp73β axis in the context of these malignancies.

## INTRODUCTION

While they are relatively uncommon cancers, Osteosarcoma and Ewing Sarcoma are the two most frequent types of Primary Bone Sarcomas, affecting both children and young adults. Resulting from a bone-remodeling processes' deregulation, these aggressive tumors mainly arise at the long bones of the extremities, and are characterized by an ectopic and anarchic osteoid neo-formation associated with osteolysis. With an incidence of 4 to 5 cases per million and a peak around 18 years old, Osteosarcoma is by far the most common type of these neoplasms [1, 2]. Although no specific genetic lesions have been uncovered until now to distinguish these tumors, the locus of the tumor suppressor *p53* gene is altered in about 50% of the patients [3, 4]. With approximately 225 new cases diagnosed per year in the United-States, Ewing Sarcoma is the second most common Bone cancer after Osteosarcoma [5]. It displays an incidence peak around 15 years of age and a slight prevalence in males [6, 7]. In 85% of the cases, Ewing Sarcoma is outlined by the chromosomal translocations t(11;22)(q24;12), giving rise to the chimeric transcription factor EWS-Fli1, whose the oncogenic features are well documented [8]. In addition, a mutational hot-spot of *p53* was suggested in Ewing Sarcoma, as the same missense mutation at codon 176 was found in several samples from primary tumors [9].

Presently, the standard of care for young patients suffering from Bone Sarcomas is based on a multimodal therapy including neo-adjuvant chemotherapy and surgical resection, together with local radiotherapy and adjuvant chemotherapy [5, 10]. Such treatments have markedly improved the outcomes of the patients worldwide, since the 5-year survival rates after treatment approach 60-70% for the localized forms. Unfortunately, chemoresistance, tumor burden and pulmonary metastases at the time of diagnosis all confer a very poor prognosis, and survival rates drop to around 30% in these cases [11]. The unsatisfactory outcomes for such patients and the toxicity -based limitations of current chemotherapeutic agents both underscore the urgency of finding novel therapeutic strategies. In this context, there is a real need to better understand the relevant drug-induced cell death processes and chemoresistance-related mechanisms.

The *P73* gene is a member of the P53-related transcription factor family. It plays a crucial role during embryonic development and tumor progression, through mechanisms involving control of the genome stability and chemosensitivity [12–17]. Although P53 and P73 display a common architecture, with several highly homologous domains, different promoters and alternative splicings contribute to the generation of a considerable number of distinct P73 isoforms. Longer isoforms bearing the trans-activating N-terminal domains are called the TA isoforms and mimic the tumor-suppressor function of p53 through their ability to trans-activate apoptotic transcriptional target-genes such as *p21, Noxa* or *PUMA* [18]. On the contrary, the ΔNp73 isoforms, lacking the TA domain, have a rather dominant-negative function [19]. Although *P53* is mutated or inactivated in about 50% of the human cancers, it is rarely the case for *P73*, making it a potent drug-induced-cell-death mediator [20, 21]. It is thus noteworthy that the expression of *P73* modulates chemosensitivity of several cancer types. Nonetheless, this feature remains to be investigated in the Bone Sarcomas' context [22].

It has already been established that miRNA-193a-5p is implicated in *P73* regulation [23]. miRNAs are small non-coding RNAs involved in the post-transcriptional regulation of the gene expression. As they act as key regulators of multiple target-genes, they are able to fine-tune various physiological processes and are aberrantly deregulated in several diseases including cancers [24–26]. These features together with their substantial stability could therefore make them potent bio-markers, novel targets or powerful drugs [27]. We have already described that the TAp73β isoform is a mediator of Cisplatin-induced apoptosis in a head and neck squamous cell carcinoma model [23]. Additionally, we also demonstrated that through modulating the expression of TAp73β, the miR-193a-5p was a key component of an endogenous Cisplatin-chemoresistance mechanism. In this study, we sought to determine if the TAp73β/miR-193a-5p axis could also be implicated in the Cisplatin-resistance of Bone Sarcomas. We observed that inhibiting TAp73β reduces the caspase activity and increases both the clonogenic features and the cell's Cisplatin-resistance. Moreover, blocking the Bone Sarcoma cells' endogenous miR-193a-5p expression reverses such effects, leading to Cisplatin-sensitization. Such results shed light on the role of the miR-193a-5p in the Bone Sarcomas' Cisplatin-chemoresistance and open the road to new therapeutic opportunities provided by its targeting in an attempt to improve the outcome of these pediatric cancers.

## RESULTS

### Human Bone Sarcoma cells express TAp73β and the miR-193a-5p and are Cisplatin- sensitive

**Figure 1:**
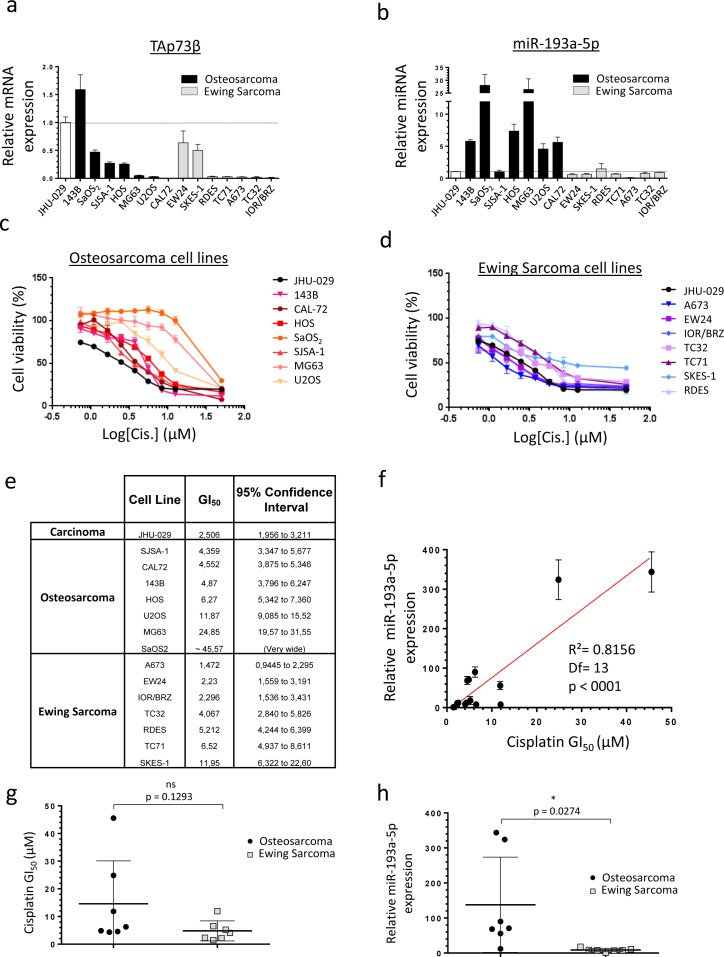
Human Bone Sarcoma cells express TAp73β and the miR-193a-5p and are Cisplatin- sensitive **a.** Expression of TAp73β and **b.** miR-193a-5p were evaluated by qRT-PCR in seven human Osteosarcoma (black patterns) and seven Ewing Sarcoma (grey patterns) cell lines and compared with the ones of the human head and neck squamous carcinoma cells JHU-029 (white pattern). Glyceraldehyde-3-phosphate dehydrogenase, β2-microglobulin and RNU6B were used as housekeeping genes. Error bars show standard deviation for *n* = 3 measurements from representative experiments. **c.** Seven human Osteosarcoma cell lines (143B, CAL-72, HOS, SaOS2, SJSA-1, MG63 and U2OS) and **d.** seven Ewing Sarcoma cell lines (A673, EW24, IOR/BRZ, TC32, TC71, SKES-1 and RDES) were cultured for 48 h in the presence of Cisplatin at the indicated concentrations and cell viability was determined by WST-1 assay. The head and neck squamous cell carcinoma cell line JHU-029 was used as a reference cell line. The viability of the non-treated control of each cell line was assigned as 100%. **e.** GI_50_ for Cisplatin in tumor cell lines. **f.** Correlation between the Cisplatin GI_50_ and the miR-193a-5p expression in the human Bone Sarcoma cell lines, assessed by WST-1 assay and by qRT-PCR and tested by the *Pearson product-moment correlation test*. **g.** GI_50_ for Cisplatin in Osteosarcoma (black patterns) and Ewing Sarcoma cell lines (grey patterns). **h.** miR-193a-5p's expression in Osteosarcoma (black patterns) and Ewing Sarcoma cell lines (grey patterns). An unpaired Student's *t-test* was used to compare the differences between the mean values of the Cisplatin-GI_50_ and the miR-193a-5p's expression in the Osteosarcoma and in the Ewing Sarcoma cell lines.

To directly assess the relevance of studying the TAp73β/miR-193a-5p axis in the drugs-resistance mechanisms of Bone Sarcomas, we first evaluated the expression of TAp73β at mRNA level and the miR-193a-5p's expression in seven Osteosarcoma cell lines and in seven Ewing Sarcoma cell lines (Figure 1a, 1b). As the JHU-029 head and neck squamous carcinoma cells were previously used as a model to study the p63/miR193a-5p/p73 axis, they were included in our screening and serves as a control [23]. All the cell lines express TAp73β except the CAL-72 one, potentially because it is the only Osteosarcoma cell line of our panel displaying a wild-type and functional p53 status (Figure 1a, Supp. Figure 1). In addition, the 143B cell line displays the highest expression of this apoptotic factor. It is indeed about 50% more elevated than in the JHU-029 carcinoma cells previously presented as over-expressing it [23]. The SaOS_2_, SJSA-1, HOS, EW24 and SKES-1 cells display an intermediate expression level, whereas this transcription factor is barely detectable in the other cell lines. The miR-193a-5p is expressed in all the cell lines tested, but significantly more in the Osteosarcoma cells compared to the Ewing Sarcoma ones (Figure 1b). These results strongly suggest the implication of the miR-193a-5p in Osteosarcoma-specific cellular processes but nevertheless fully justify pursuing this work in both Bone Cancer types in order to assess the impact of such discrepancy in the drug-resistances' context. The same Bone Sarcoma cells' panel was treated with Cisplatin to assess its effects on the cell viability (Figure 1c, 1d, 1e). A concentration-dependent inhibition of cell viability was observed in all the cell lines studied with important variability of the GI_50_. The Ewing Sarcoma cells A673, EW24 and IOR/BRZ are the more sensitive ones as they exhibited GI_50_ between 1.472 and 2.296 μM (Figure 1d, 1e). With GI_50_ comprise between 4.067 and 11.95 μM, the SJSA-1, CAL-72, 143B, HOS, U2OS, TC32, RDES, TC71 and SKES-1 cells display an intermediate sensitivity. In contrast, the MG63 and SaOS_2_ cell lines are twenty to forty times less sensitive compared with the first ones, with a GI_50_ of 24.85 μM and around 45.57 μM respectively. Interestingly, those two cell lines are the ones displaying the highest miR-193a-5p levels (Figure 1b). A statistically significant correlation was found between the GI_50_ and the miR-193a-5p expression level in all the cell lines, reinforcing the hypothesis of the miR-193a-5p's involvement in the Cisplatin-chemoresistance in such model (Figure 1f). In addition, a non-significant higher global average GI_50_ is observed in the Osteosarcoma compared with the Ewing Sarcoma cells (Figure 1g). Interestingly, a significant higher global expression of the miR was also found in the Osteosarcoma cell lines compared with the Ewing Sarcoma ones (Figure 1h), arguing again in favor of its presumed implication in the Cisplatin-chemoresistance. Following investigations were thus based on the hypothesis that a high miR-193a-5p expression could induce a high Cisplatin-chemoresistance due to the miRNA-inhibitor's effects on TAp73β's expression. The cell lines were thus chosen for further analysis based on their TAp73β and miR-193a-5p expression levels.

### Cisplatin modulates the expression level of the miR-193a-5p, TAp73β and its target-genes in Bone Sarcoma cells

**Figure 2:**
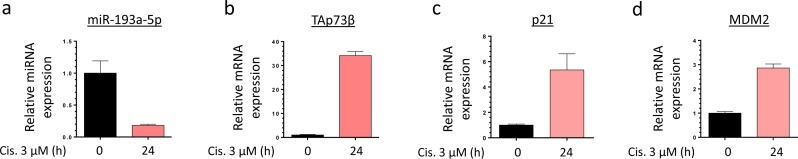
Cisplatin modulates the expression level of the miR-193a-5p, TAp73β and its target-genes in Bone Sarcoma cells Expression of miR-193a-5p **a.**, TAp73β **b.**, p21 **c.** and MDM2 **d.** were evaluated by qRT-PCR in the RDES Ewing Sarcoma cell line after treating the cells or not during twenty-four hours with 3 μM Cisplatin. RNU6B, Glyceraldehyde-3-phosphate dehydrogenase and β2-microglobulin were used as housekeeping genes. Error bars show the standard deviation for *n* = 3 measurements from representative experiments.

To better understand to what extent the miR-193a-5p/TAp73β axis is involved in the Cisplatin-response of the Bone Sarcoma cells, the RDES Ewing Sarcoma cell line and the SJSA-1 Osteosarcoma one were treated or not with 3 μM Cisplatin during twenty-four hours and the expression levels of the miR-193a-5p, TAp73β and two of its target-genes, p21 and MDM2 were assessed by qRT-PCR (Figure 2a, 2b, 2c, 2d and Supp. Figure 2a, 2b, 2c, 2d). In the RDES cells, we can notice that the Cisplatin induces a down-regulation of the miR-193a-5p from about 82% compared with the non-treated control cells. As expected, the expression levels of TAp73β and two of its direct targets are strongly increased in these conditions. Similar results were observed in the SJSA-1 cells except for MDM2 expression (Supp. Figure 2a, 2b, 2c, 2d). Those two cell lines were picked because of their relatively low TAp73β expression level allowing and easier activation observation. These results highlight the inhibitory effect of the Cisplatin on the expression of the miR-193a-5p, but the exact underlying mechanism needs to be investigated.

### TAp73β-mediated Cisplatin sensitivity of human Bone Sarcomas

**Figure 3:**
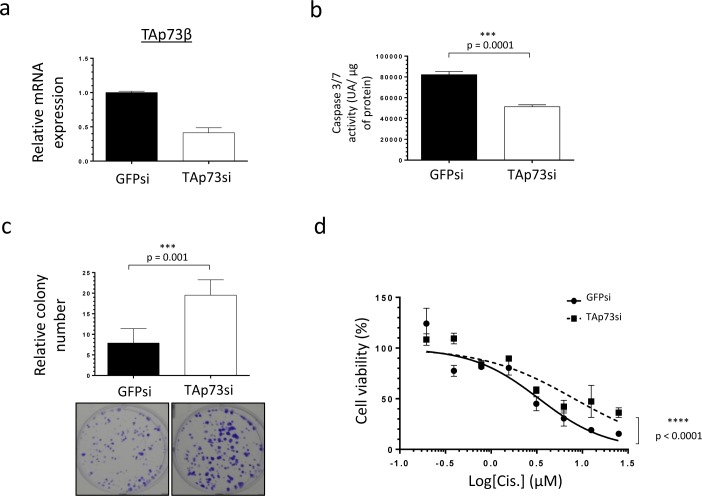
TAp73β-mediated sensitivity of human Bone Sarcomas: regulation of apoptosis and clonogenicity **a.** Expression of TAp73β was evaluated by qRT-PCR in the RDES Ewing Sarcoma cell line, after infecting the cells with viral-supernatant of GFPsi -or TAp73si-transduced HEK293FT cells. Glyceraldehyde-3-phosphate dehydrogenase and β2-microglobulin were used as housekeeping genes. **b.** The basal apoptosis level was then evaluated by dosage of the caspase 3/7 activity in protein extracts from the same cells and in the same conditions as in (a). Error bars show the standard deviation for *n* = 3 measurements from representative experiments. **c.** The basal clonogenic capabilities of the cells were evaluated in the same cells and in the same conditions as in (a). One thousand cells were seeded in 6-wells plates and incubated until the possibility of macroscopic clones counting. The cells were then fixed in glutaraldehyde and stained with Crystal Violet. Error bars show the standard deviation for *n* = 3 measurements from representative experiments. Representative pictures of the wells in each condition were chosen. An unpaired Student's *t-test* was used to compare the different conditions in the caspase 3/7 activity assays and in the clonogenic assays. **d.** The same cells in the same conditions as in (a) were cultured for 48 h in the presence of Cisplatin at the indicated concentrations and cell viability was determined by WST-1 assay. The viability of the non-treated control of each cell line was assigned as 100%. A two-way ANOVA test was used to compare the different conditions in the viability assays.

In order to better delineate to what extent TAp73β is implicated in the Cisplatin-response of Bone Sarcoma cells, TAp73 was down-regulated with siRNAs in the RDES Ewing Sarcoma cell line used in Figure 2 (with a low TAp73β expression level) and in the 143B Osteosarcoma one because they express the highest TAp73β level of all the cell lines tested (Figure 3a and Supp. Figure 3a). According to the tumor suppressive functions of TAp73 [28], the consequent 50% reduction of TAp73β's expression obtained in the RDES cell line (Figure 3a) leads to a significant diminution of its caspase 3/7 activity, supporting the implication of the TAp73β isoform in the apoptotic processes in these cells (Figure 3b). Moreover, these results are linked to the improved clonogenic capabilities observed in these conditions, as the TAp73si-cells were able to form about 2.5 times more clones in 2D than the GFPsi ones (Figure 3c). In addition, the implication of TAp73 in the Cisplatin-sensitivity of the RDES cells was highlighted by the fact that a reduced-TAp73 expression in those cells contributes to reduce their sensitivity to Cisplatin in a viability assay (Figure 3d). The same effects were confirmed in the 143B Osteosarcoma cells after TAp73 inhibition, even if this cell line displays the highest TAp73β expression level compared with the other cell lines (Supp. Figure 3a, 3b, 3c, 3d and Figure 1a). Together, these data support the implication of TAp73 in both the apoptotic and the clonogenic processes, revealing that this gene is related to the Cisplatin-sensitivity in the Bone Sarcoma cells. These results raise the question of the implication of the miR-193a-5p in the Cisplatin-chemoresistance though its ability to regulate TAp73β.

### The TAp73β's targeting miR-193a-5p is implicated in the Cisplatin chemoresistance of human Bone Sarcomas

**Figure 4:**
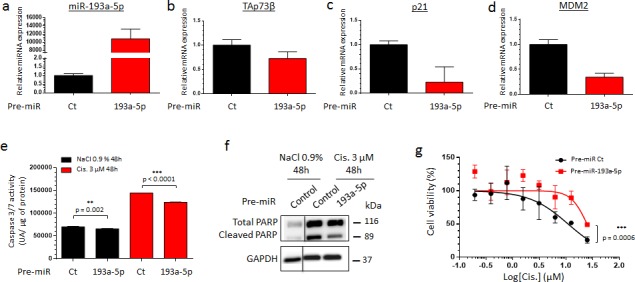
The TAp73β's targeting miR-193a-5p is implicated in the Cisplatin chemoresistance of human Bone Sarcomas **a.** miR-193a-5p expression was assessed by qRT-PCR in the RDES Ewing Sarcoma cell line forty-eight hours after either the pre-miR control or the pre-miR-193a-5p mimic's transfection. The expression of TAp73β **b.**, p21 **c.** and MDM2 **d.** were assessed by qRT-PCR in the same conditions as described in (a). RNU6B, Glyceraldehyde-3-phosphate dehydrogenase and β2-microglobulin were used as housekeeping genes for qRT-PCR and the error bars show the standard deviation for *n* = 3 measurements from representative experiments. **e.** RDES Ewing Sarcoma cell line was transiently transfected in the same conditions as in (a) and was treated forty-eight hours later with 3 μM Cisplatin or the same amount of NaCl 0.9% for additional forty-eight hours. The apoptosis was then evaluated by dosage of the caspase 3/7 activity in protein extracts. Error bars show the standard deviation for *n* = 3 measurements from representative experiments. A two-tailed paired Student's *t-test* was used to compare the different conditions in the caspase 3/7 activity assays. **f.** Protein's extracts from RDES Ewing Sarcoma cell line in same conditions as in (a) were subjected to Immunoblotting with anti-cleaved-PARP antibodies. Glyceraldehyde-3-phosphate dehydrogenase was used as loading control. **g.** RDES Ewing Sarcoma cell line was transiently transfected with either pre-miR control or pre-miR-193a-5p mimics and was cultured forty-eight hours later in the presence of Cisplatin at the indicated concentrations for two hours. The cell viability was determined by WST-1 assay and compared with control. The viability of the non-treated control was assigned as 100%. A two-way ANOVA test was used to compare the different conditions in the viability assays. Data refer to three different experiments and western blot images are representative of these. Black lines show where the original gel was cropped to obtain the final image

To assess the impact of the miR-193a-5p in the Cisplatin-resistance, its overexpression was performed in the RDES Ewing Sarcoma cell line and in the SJSA-1 Osteosarcoma one. These two cell lines display a non-functional p53 protein, weakly express both TAp73β and the miR-193a-5p and exhibit the same intermediate Cisplatin sensitivity as shown by their equivalent GI_50_ (Figure 1 and Supp. Figure 1). Such features raise the opportunity to easily increase the miR-193a-5p's expression level by transient transfections of pre-miRNAs and make them interesting models in the context of our study. The pre-miR-193a-5p was thus transiently transfected and the cells were treated forty-eight hours later with Cisplatin (Figure 4 and Supp. Figure 4). The transfection's efficiency was validated by qRT-PCR in both cell lines (Figure 4a and Supp. Figure 4a). The consequent expected down-regulation of TAp73β, p21 and MDM2 was also verified at transcriptional level (Figure 4b, 4c, 4d and Supp. Figure 4b, 4c, 4d). In absence of Cisplatin, increasing the miR-193a-5p's expression induces a slight reduction in the caspase 3/7 activity (Figure 4e and Supp. Figure 4e), corroborating the previous results obtained after reducing the expression of TAp73 (Figure 3b and Supp. Figure 3b). The same effect is observed after a 3 μM Cisplatin-treatment. Increasing the miR-193a-5p's expression significantly reduces the caspase 3/7 activity by 14.13 and by 13.23% in the RDES and in the SJSA-1 cells respectively. Inducing the expression of the miR-193a-5p also reduces the Poly-(ADP-ribose) polymerase (PARP) cleavage induced by the Cisplatin (Figure 4f and Supp. Figure 4f) and improves the cell viability after only two hours of Cisplatin treatment in both cell lines (Figure 4g and Supp. Figure 4g). Taking together, these results argue for the anti-apoptotic role of the miR-193a-5p, through its TAp73β's targeting capabilities and strongly support its implication in the Cisplatin-chemoresistance in this model.

### The TAp73β-mediated Cisplatin-induced cell death is countered by miR-193a-5p in human Bone Sarcoma cells

**Figure 5:**
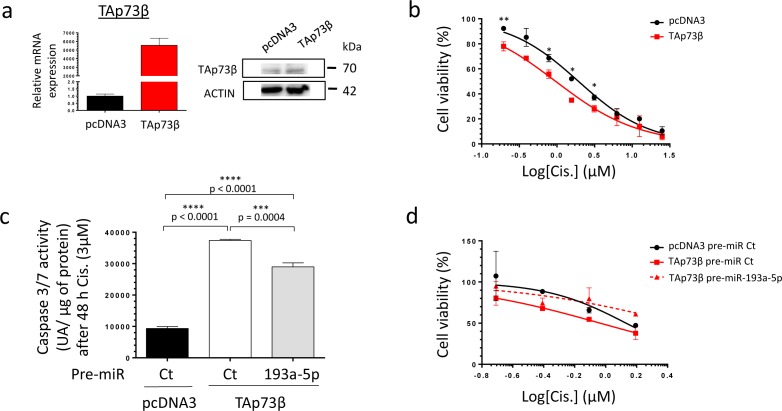
The TAp73β-mediated Cisplatin induced cell death is opposed by the miR-193a-5p in human Bone Sarcoma cells **a.** The TAp73β's expression was assessed in the MG63 Osteosarcoma cell line both at mRNA level by qRT-qPCR (left panel) and at protein level by Western Blotting (right panel) forty-eight hours after the cell's transient transfection with the empty vector pcDNA3 or the TAp73β one. Glyceraldehyde-3-phosphate dehydrogenase and β2-microglobulin were used as housekeeping genes for qRT-PCR. Actin was used as a loading control for Immunoblotting. **b.** MG63 Osteosarcoma cell line was transiently transfected with either the empty vector pcDNA3 or the TAp73β one and was cultured forty-eight hours later in the presence of Cisplatin at the indicated concentrations for four days. The cell viability was determined by WST-1 assay and compared with control. The viability of the non-treated control was assigned as 100%. Error bars show the standard deviation for *n* = 2 measurements from representative experiments. An unpaired Student's *t-test* was used to compare the different conditions in the viability assays. **c.** MG63 cells were transiently transfected with either the pre-miR control or the pre-miR-193a-5p as the same time as pcDNA3 empty vector or the TAp73β containing-one. The cells were then cultured forty-eight hours later in the presence of 3 μM Cisplatin for additional forty-eight hours. The apoptosis was then evaluated by dosage of the caspase 3/7 activity in protein extracts. Error bars show the standard deviation for *n* = 3 measurements from representative experiments. A two-tailed paired Student's *t-test* was used to compare the different conditions in the caspase 3/7 activity assays. **d.** MG63 cells were transiently transfected with either the pre-miR control or the pre-miR-193a-5p as the same time as pcDNA3 empty vector or the TAp73β containing-one. The cells were then cultured forty-eight hours later in the presence of 3 μM Cisplatin at the indicated concentrations for additional forty-eight hours. The cell viability was determined by WST-1 assay and compared with control. The viability of the non-treated control was assigned as 100%.

To address the ability of miR-193a-5p to modulate the Cisplatin sensitivity of Bone Sarcoma cells through targeting TAp73β, a modulation of the expression level of both partners was performed. The MG63 cells display one of the highest basal Cisplatin chemoresistance, probably due to their elevated miR-193a-5p's expression level and their weak basal TAp73b's expression level compared with all the Bone Sarcoma cell lines previously screened (Figure 1). Regarding those features, this cell line appears as the best model to follow this strategy. To assess the implication of TAp73β in the Cisplatin-chemoresistance in this cell line, the cells were first transiently transfected with a TAp73β coding vector only. The efficiency of the transfection was validated by qRT-PCR, as the expression of TAp73β was about five thousand times more elevated in the TAp73β-transfected cells than in the control ones (Figure 5a left panel). These results were also confirmed at protein level, however in a lower extent (Figure 5a right panel). Such increase has a functional effect on the cell viability, increasing the cell sensitivity in response to a four-days Cisplatin treatment at each concentration tested (Figure 5b). Moreover, increasing the expression of TAp73β markedly improve the Cisplatin-induced caspase 3/7 activity (Figure 5c). Nonetheless, increasing the expression of the TAp73β-targeting miR-193a-5p significantly decreases the caspase 3/7 activities by 22.5%. Finally, as predicted by those data, increasing the expression of the miR-193a-5p counteracts the Cisplatin-chemo-sensitizing effects of TAp73β on the cell viability (Figure 5d).

### Inhibiting the miR-193a-5p increases the expression of TAp73β and restores the Cisplatin-sensitivity of human Bone Sarcoma cells

**Figure 6:**
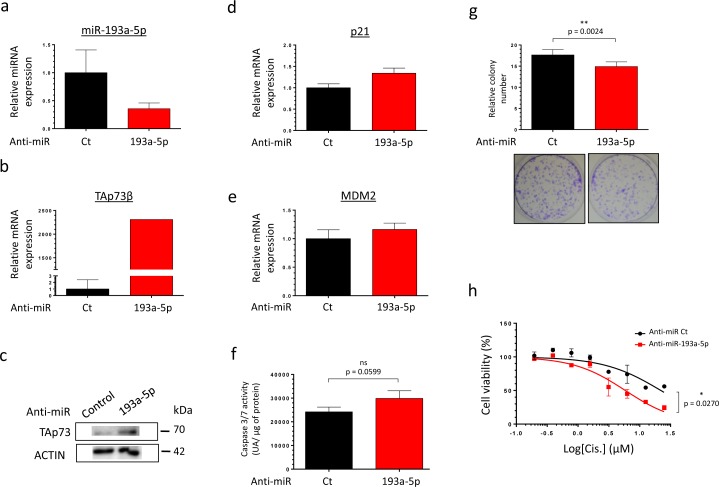
Inhibiting the miR-193a-5p increases the expression of TAp73β and restores the Cisplatin-sensitivity of human Bone Sarcoma cells **a.** miR-193a-5p's expression was assessed by qRT-PCR in the MG63 Osteosarcoma cell line forty-eight hours after either the anti-miR control or the anti-miR-193a-5p's transfection. TAp73β's expression was assessed by qRT-PCR **b.** and by Western Blotting **c.** in the same conditions as described in (a). Glyceraldehyde-3-phosphate dehydrogenase and β2-microglobulin were used as housekeeping genes for qRT-PCR and actin serves as Immunoblotting's loading control. The expression of p21 **d.** and MDM2 **e.** were assessed by qRT-PCR in the same conditions as described in (a). β2-microglobulin was used as housekeeping genes for qRT-PCR. For the qRT-PCR experiments, error bars show the standard deviation for *n* = 3 measurements from representative experiments. **f.** The basal apoptosis level was then evaluated by dosage of the caspase 3/7 activity in protein extracts from the same cells and in the same conditions as in (a). Error bars show the standard deviation for *n* = 3 measurements from representative experiments. **g.** The basal clonogenic capabilities of the cells were evaluated in the same cells and in the same conditions as in (a). One thousand cells were seeded in 6-wells plates and incubated until the possibility of macroscopic clones counting. The cells were then fixed in glutaraldehyde and stained with Crystal Violet. Error bars show the standard deviation for *n* = 3 measurements from representative experiments. Representative pictures of the wells in each condition were chosen. A two-tailed paired Student's *t-test* was used to compare the different conditions in the caspase 3/7 activity assays and in the clonogenic assays. **h.** MG63 Osteosarcoma cell line was transiently transfected with either the anti-miR control or the anti-miR-193a-5p and was cultured forty-eight hours later in the presence of Cisplatin at the indicated concentrations for two hours. The cell viability was determined by WST-1 assay and compared with control. The viability of the non-treated control was assigned as 100%. A two-way ANOVA test was used to compare the different conditions in the viability assays.

As TAp73β is a target of the miR-193a-5p, we hypothesized that decreasing the endogenous expression level of this miR may have the same consequences on the Cisplatin-sensitivity than over-expressing TAp73β. We then tested our sensitizing strategy on the partially Cisplatin-resistant cell line MG63, moreover expressing high level of miR-193a-5p. The MG63 cells were thus transfected with a control anti-miR or the anti-miR-193a-5p and the efficiency of transfection was validated by qRT-PCR (Figure 6a). As expected, the expression of TAp73β and two of its target-genes (p21 and MDM2) was increased (Figure 6b, 6c, 6d, 6e). The functional consequence of such modulation was assessed on the caspase 3/7 activities and on the clonogenic capabilities of those cells (Figure 6f, 6g). Without Cisplatin-treatment, inhibiting the expression of the miR only leads to a slight non significant increase of the caspase activity (Figure 6f) but significantly decreases the cell colony-forming ability (Figure 6g). In addition, inhibiting the expression of the miR-193a-5p potentiates the effects of the Cisplatin on the cell viability, significantly sensitizing the cells to this agent (Figure 6h). In addition, the use of the anti-miR-193a-5p also displays a chemo-sensitizer's effect both on the 143B Osteosarcoma and the RDES Ewing Sarcoma cells, in which the expression of TAp73β was artificially reduced (Supp. Figure 5a and b). Taken together, these results bring the proof of concept that artificially inhibiting the miR-193a-5p expression could be a useful strategy to potentiate the Bone Sarcoma cell's sensitivity to the Cisplatin.

## DISCUSSION

The chemoresistance developed by the tumor's cells is one of the main causes of the cancer-therapies' failure, especially in Bone Sarcomas which are very aggressive and in which only few therapeutic options are available. The origins of the acquired drug-resistance can be multiple, but a progressive loss of expression of tumor-suppressor genes along the treatment-course often occurs in cancer cells. As these genes normally sense the genotoxic stress or mediate the drug-induced apoptotic response, such cancer cells can overcome the chemotherapeutic agents' effects. Beyond the mutational processes that can affect such tumor-suppressor's expression or activity, their silencing by the miRNAs is an epigenetic mechanism that has been well accepted to deregulate the drug-resistance-related genes. Unfortunately, only a few studies are available in the Bone Sarcoma's context and the precise functions of these molecules still need to be deciphered. For instance, the miR-34c was reported to be decreased in Cisplatin-poor responders' Osteosarcoma patients compared to good ones^51^.

Here, we report that the p53-related tumor-suppressor gene TAp73β is implicated in the Cisplatin-induced apoptosis of Bone Sarcomas and that the miRNA-193a-5p, through its TAp73β's targeting ability, consequently modulates the Cisplatin-sensitivity of these cancers. Interestingly, we have highlighted that this mechanism occurs both in Osteosarcoma and Ewing Sarcoma, even if the global expression levels of TAp73β and the miRNA-193a-5p are lower in Ewing Sarcoma (Figure 1a, 1b). The difference in the global expression level of TAp73β between these two models might be explained by the osteoblastic-origin of Osteosarcoma and by the requirement of p73 during the vitamin D-mediated osteoblastic differentiation [29]. In addition, this difference could also be supported by the fact that human bone marrow mesenchymal stem cells, which are the cells of origin of Ewing Sarcoma, display a silenced-expression of the *p73* gene through the hypermethylation of its promoter [30]. Furthermore, according to both its p53-related role and pro-apoptotic functions, the expression of TAp73 is often higher in a p53-functionnally deficient cellular-background [31]. This is in agreement with the wild-type p53 status of the CAL-72 Osteosarcoma cell line, in which no TAp73β expression was detectable (Figure 1a and Supp. Figure 1). In addition, even if the miRNA-193a-5p is poorly documented in the Bone context, the expression of this miR appears to decrease during the osteogenic differentiation of human adipose-derived stem cells [32]. However, the miR-193a-3p was reported to be five times more elevated in the MG63 Osteosarcoma cell line than in the HOB osteoblasts, letting presuming here of its oncogenic role [33].

Additionally, this study reports for the first time the inhibitory effect of the Cisplatin on the miR-193a-5p's expression of Bone Sarcomas. As a consequence, the expression of the pro-apoptotic factor TAp73β and two of its target-genes, p21 and MDM2 are increased (Figure 2 and Supp. Fig. 2). Our results are in accordance with a previous work, demonstrating that increasing TAp73β's expression leads to a rapid and robust up-regulation of p21 in the SaOS_2_ Osteosarcoma cell line [34]. Through overexpression and knockdown experiments, we found that TAp73β is implicated in the regulation of both the caspase 3/7 activity and the clonogenic capabilities of the Bone Sarcoma cells. Thus, such involvement in these cellular processes functionally confers to TAp73β a mediator role in the Cisplatin-induced apoptosis of the Bone Sarcomas. These results corroborate recent studies, which have also demonstrated the role of p73 in the Cisplatin-related apoptosis in several models [35, 36].

Regarding its p53-redundant-functions, the epigenetic regulation of TAp73β through miRNAs has already been described and interestingly, even if *in silico* analysis predicts that the miRs -125b, -486-3p and -34a could potentially target TAp73 [37], only the miR-193a-5p was validated as a direct *bona-fide* TAp73β's repressor [23]. Through modulating the miR-193a-5p's expression level, we highlighted its implication in the Cisplatin-chemoresistance of the Bone Sarcoma cells, as previously shown in a head and neck squamous cell carcinoma model [23]. In contradiction with our results (Figure 4e, 5c, 6f and Supp. Figure 4e), it was interestingly previously reported that the miR-193 is an inducer of the caspase-3 activity, arguing here for its tumor-suppressive functions [38]. In addition, beyond the fact that the miR-193a-5p can probably inhibit multiple target-genes, it seems clear that its dual role in the apoptotic-processes' control could be partially explained by its p73-targeting features. Obviously, the different functions of the isoforms of p73 are not yet fully elucidated and are undoubtedly modulated by the interactions of p73 itself with the other members of the p53 family, especially p63. In return, this gene-family also regulates the expression of this miRNA, as it was reported that the oncogenic transcription factor ΔNp63α is an inducer of the miR-193a-5p's expression, thus contributing to the Cisplatin-chemoresistance [23]. These data could explain a previous study reporting that a stable p73 knock-down in breast cancer cell lines induce a higher Cisplatin chemoresistance in the cells which express ΔNp63 compared with these which do not [36].

In summary, we present here evidence for an original epigenetic regulatory mechanism placing the TAp73β/miRNA-193a-5p axis as a major pathway contributing to the Cisplatin-sensitivity of the Bone Sarcomas. From a therapeutic standpoint, our study highlights the proof-of-concept that inhibiting the miR-193a-5p expression consequently sensitizes the Bone Sarcoma cells to the Cisplatin. Finally, this work sustains the relevant feasibility of using miRNAs-inhibitors in association with standard Cisplatin-treatment to improve the response of the young patients treated with this drug.

## MATERIALS AND METHODS

### Tumor cell lines and therapeutic agents

Seven human Ewing Sarcoma cell lines were used: the A673 TC32, SKES-1 and RDES cell lines, which were kindly provided by Dr. S. Burchill (Children's Hospital, Leeds, United Kingdom), the EW24 and TC71 cell lines, which were a gift from Dr. O. Delattre (Institut National de la Santé et de la Recherche Médicale U830, Paris, France) and the IOR/BRZ one. Seven Osteosarcoma cell lines were studied: 143B, CAL-72, MNNG/HOS (thereafter called HOS), SaOS_2_, SJSA-1, MG63 and U2OS. All these cells provide from the American Tissue Cell Collection (ATCC). The JHU-029 cells were a generous gift of David Sidransky, MD (Johns Hopkins University, USA) and are used as positive control because of their use in the previous study of Ory et al. [23]. HEK 293FT cells (Invitrogen, Life Technologies, Carlsbad, CA, USA) are used to produce lentiviral particles. See supplementary methods for detailed cell culture conditions. The Cisplatin powder was provided by Sigma and solubilized in 0.9% NaCl solution and stored at −20°C.

### Lentiviral and retroviral production

P73-shRNA lentiviral particles were produced by transfection of required viral plasmids (4 μg of each: RSV-RRE, RGR, VSV-G and 4 μg of TAp73-shRNA; TAp73si; or 4μg of GFP-shRNA; GFPsi) following the manufacturer's protocol (CalPhos Transfection Kit; Clontech) in HEK 293 FT cell line. The 143B, and the RDES cells were infected with HEK 293T supernatants (with GFPsi as a control or TAp73si) and selected by antibiotic resistance (puromycin, 1μg/mL for the 143B cells or 0.5 μg/mL for the RDES ones) for several weeks. P73-shRNA targeted sequence is available upon request.

### Transient transfection of pre-miR^TM^ and anti-miR^TM^

All the pre-miR^TM^ and anti-miR^TM^ were obtained from Ambion and transfected at a final concentration of 30 nM using the siPORTNeoFX Transfection Agent (Ambion) according tothe manufacturer's protocol.

### Transient transfection of TAp73β

The TAp73β sequence was inserted in pcDNA3 vector (Invitrogen). 2μg of DNA were transfected using FuGENE® HD Transfection Reagent (Promega) according to the manufacturer's protocol. These assays were all performed as described [23].

### Cell viability assay and GI_50_ calculation

Bone Sarcoma cell lines were plated in 96-wells plates in the appropriate medium with 10% FBS and treated with Cisplatin at indicated concentration during 48 hours and cell growth was measured using the WST-1 assay (Roche, Mannheim, Germany). At the end of the treatment time, the culture medium is removed and replaced by the WST-1 reagent diluted in fresh medium in a 1:10 proportion. The absorbance at 470 nm was measured on a 96-multiwell microplate reader (Victor^2^ 1420; PerkinElmer Inc.) and normalized to the average reading of wells containing medium only. The GI_50_ were calculated thanks to the GraphPad Prism 6 software.

### 2D clonogenic assay

Bone Sarcoma cells were seeded at a density of 1000 cells per well in 6-wells plate and treated with 3 μM of Cisplatin or the corresponding amount of NaCl 0.9% for 48 hours. Culture medium was then replaced by fresh one and the cells were cultured for additional 6 days. The colonies were then washed in PBS, fixed with glutaraldehyde 10% and stained with Crystal Violet (1% in water). Pictures were taken and five areas/well were arbitrary chosen to represent the entire surface of each well. The stained surfaces/total surfaces of each area/well were calculated thanks to the ImageJ software.

### Apoptotic-cell death assessment

The caspase 3/7 activity was assessed thanks to the apo-ONE® Homogeneous caspase-3/7 Assay kit (Promega, Madison, USA) according to the manufacturer's instructions.

### Total RNA extraction and quantitative reverse transcription-PCR

Total RNA was extracted from cultured-cells using the QIAzol Lysis Reagent (QIAGEN) and the miRNeasy Mini Kit (QIAGEN) according to the manufacturer's instructions. Total RNA was reversed transcribed using the ThermoScript RT-PCR System (Life Technologies). Real-time monitoring of PCR amplification was performed on CFX96 real-time PCR detector system (Bio-Rad, Marnes la Coquette, France) with SYBR PCR Master Mix buffer (Bio-Rad). Target gene expression was normalized to glyceraldehyde 3-phosphate dehydrogenase (GAPDH), and β-2 microglobulin (B2M) levels in respective samples as an internal standard, and the comparative cycle threshold (Ct) method was used to calculate the relative amplification of target messenger RNAs. The primers sequences are described in the Supp. Table 2.

### Quantitative reverse transcription-PCR for miRNAs

A specific RT was performed for each miRNA from 100 ng of total RNA, using specific stemloop RT primers (50 nM) and the MultiScribe Reverse transcriptase (Applied Biosystems). The RT conditions were as follows: 30 minutes at 16°C followed by 30 seconds at 20°C, 30 seconds at 42°C, 1 second at 50°C for 60 cycles, and finally 5 minutes at 85°C. The expression of each gene was normalized to the one of the small nuclear U6B RNA as a reference. The miRNA's RT-qPCR-primers' sequences are detailed in Supp. Table 3 and 4.

### Western blotting analysis

Samples containing equal amounts of protein (depending on the antibody, 15-80 mg) from lysates of cultured Bone Sarcoma cell lines underwent electrophoresis on SDS-PAGE and were transferred to polyvinylidene difluoride (PVDF) membranes. The membranes were blocked in 3% BSA-PBS-0.05% Tween at room temperature for 1 hour and blots were probed overnight at 4°C with the primary antibodies. The features of the latter are summarized in the Supp. Table 5. Membranes were then saturated 1 hour with 5% milk-PBS-0.05% Tween (Régilait) and finally incubated for 1 hour with secondary antibodies at room temperature (Supp. Table 5). Specific proteins were detected using SuperSignal® West Dura Extended Duration Substrate (ThermoScientific, Rockford, USA) and a G-Box (Syngene, Cambridge, UK) after washing. Pictures were analysed thanks to the ImageJ software.

## SUPPLEMENTARY MATERIALS


