# Supplementary material for: miRNA-193a-5p repression of p73 controls Cisplatin chemoresistance in primary bone tumors
Source: Oncotarget. 2016 Jul 29;7(34):54503–14. doi: 10.18632/oncotarget.10950 (PMC5342358; doi:10.18632/oncotarget.10950)
Supplement: Supplementary file 1 [file oncotarget-07-54503-s001.pdf]

miRNA-193a-5p repression of p73 controls Cisplatin chemoresistance in primary bone tumors

Supplementary Material

Cell culture conditions

All the cell lines were cultured in the presence of 1% penicillin/streptomycin in a humidified 5% CO_2/air atmosphere at 37 °C and were passaged for less than 3 months. and Type I collagen was required collagen (Sigma-Aldrich) was required to allow the STAET-1 and TC71 cells to grow. The HEK 293 FT cells were cultured in presence of 0.5 mg/mL Geneticine (G418, Sigma, Saint Quentin, France) to maintain the SV40-T antigen expression all cell lines were cultured. All the features and the culture conditions of the cells are summarized in the **Supp. Table 1**. Cisplatin (Sigma-Aldrich Corp., St. Louis, MO, USA) is purchased in powder form, solubilized in NaCl 0.9% at a 2 mg/mL concentration and stored at -20°C.

Statistical analysis

For each experimental data point, the standard deviation from replicate experiments was calculated as noted in the legends and is shown as error bars. All error bars show standard deviation for at least triplicate measurement from representative experiments. The mean ± s.d was calculated for all groups and compared by two-tailed paired Student's *t-test* or by ANOVA analysis of variance. $P < 0.05$ was used as the criteria for statistical significance. In the **Fig. 1f**, the Pearson product-moment correlation coefficient (R^2) was calculated and a two-tailed P-value was generated from a probability table. GraphPad Prism 6 software was used for all statistical analysis.

Origin	Cell line name	Clinical features			Culture conditions
		Gender	Age	Location	
Osteosarcoma	**143B HOS**	F	13	femur	RPMI + 10% FBS
	**CAL-72**	M	10	knee recur	DMEM + 10% FBS
	**MG63**	M	14	bone	DMEM + 10% FBS
	**MNNG-HOS**	F	13	femur	RPMI + 10% FBS
	**SaOS2**	F	11	bone	DMEM + 10% FBS
	**SJSA-1**	M	19	femur	RPMI + 10% FBS
	**U2OS**	F	15	tibia	DMEM + 10% FBS
	**A673**	F	15	muscle	DMEM + 10% FBS
Ewing Sarcoma	**EW24**	nd	nd	nd	RPMI + 10% FBS
	**IOR/BRZ**	nd	nd	nd	IMDM + 10% FBS + 2 mmol/L L-glutamine
	**RDES**	M	19	humerus	DMEM + 10% FBS
	**SKES-1**	M	18	bone	DMEM + 10% FBS
	**STAET-1**	F	13	humerus	DMEM + 10% FBS
	**TC32**	nd	17	nd	DMEM + 10% FBS
	**TC71**	M	22	humerus recur	RPMI + 10% FBS
Head and neck squamous cell carcinoma	**JHU-029**	nd	nd	nd	RPMI + 10% FBS
Kidney embryo	**HEK 293 FT**	/	/	/	DMEM + 10% FBS

Table 1: The main clinical and experimental features of cell lines used. DMEM: Dulbecco's modified Eagle's medium; IMDM: Iscove’s modified Dulbecco’s medium; RPMI: Roswell Park Memorial Institute Medium; nd: no data. The culture medium are purchased from Invitrogen-Life Technologies and the cells were cultured in presence of 10% fetal bovine serum (FBS).

Gene (human)	Forward primer (5'-3')	Reverse primer (5'-3')
TAp73β	GCACCACGTTTGAGCACCTCT	GCAGATTGAACTGGGCCATGA
B2M	AGCTGTGCTCGCGCTACTCTC	CACACGGCAGGCATACTCATC
MDM2	CCATGATCTACAGGAACTTGGTAGTA	TCACTCACAGATGTACCTGAGTCC
P21	CGAAGTCAGTTCCTTGTGGAG	CATGGGTTCTGACGGACAT
GAPDH	TGGGTGTGAACCATGAGAAGTATG	GGTGCAGGAGGCATTGCT

Table 2: Sequences of the forward and reverse primers used in the qPCR.

miRNA	Hairpin RT primer (5'-3')
RNU6B	GTCGTATCCAGTGCAGGGTCCGAGGTATTCGCACTGGATACGACAAAAATA
hsa-miR-193a-5p	GTCGTATCCAGTGCAGGGTCCGAGGTATTCGCACTGGATACGACTCATCT

Table 3: Sequences of the Hairpin RT primers used for the miRNA-RT.

miRNA	Forward primer (5'-3')	Universal Reverse primer (5'-3')
RNU6B	CGCAAGGATGACACGCAA	GTGCAGGGTCCGAGGT
hsa-miR-193a-5p	TATATGGGTCTTTGCGGGCG	GTGCAGGGTCCGAGGT

Table 4: Sequences of the different forward and universal reverse primers used in the qPCR for the miRNAs.

Name	Dilution	Provider
mouse anti-**p73**(Ab-2), ER-15 clone, Cat#OP109	1:500	CALBIOCHEM, EMD Biosciences, Inc.
rabbit anti-**PARP** #9542S	1:1000	Cell Signaling Technologies
rabbit anti-**GAPDH** 14c10	1:2000	Cell Signaling Technologies
rabbit anti-**actin** A5060 (20-33)	1:10000	Sigma-Aldrich
goat-anti-**rabbit** sc-2004 #J1512	1:10000	Santa Cruz Biotechnologies
donkey-anti-**mouse** sc-2314 #C2012	1:5000	Santa Cruz Biotechnologies

Table 5: References of the antibodies used in Western blot.

Bone Sarcoma type	Cell line	P53 status
Osteosarcoma	143B	R156P;F270L
	CAL72	wt
	HOS	R156P;F270L
	MG63	rearranged
	$SaOS_2$	null
	SJSA-1	wt inactivated
	U2OS	wt inactivated
Ewing Sarcoma	A673	T118fsX5 or A119fsX5
	EW24	K164E
	IOR/BRZ	?
	RDES	R273C
	SKES-1	C176F
	TC32	wt
	TC71	R213X

Supplementary Figure 1: p53 status of the Human Bone Sarcoma cell lines.

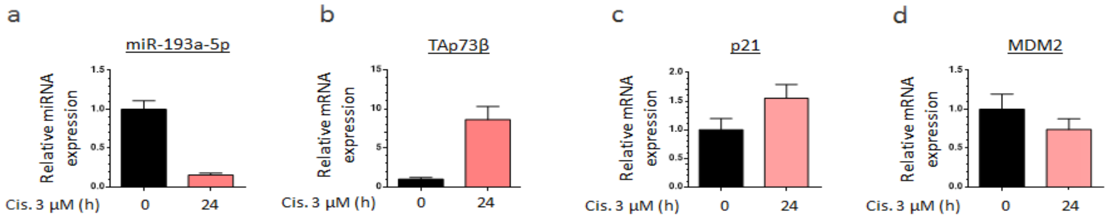

Supplementary Figure 2: Cisplatin modulates the expression level of the miR-193a-5p, TAp73β and its target-genes in Bone Sarcoma cells.

Expression of miR-193a-5p (**a**), TAp73β (**b**), p21 (**c**) and MDM2 (**d**) were evaluated by qRT–PCR in the SJSA-1 Osteosarcoma cell line after treating the cells or not during twenty-four hours with 3 µM Cisplatin. RNU6B, Glyceraldehyde-3-phosphate dehydrogenase and β2-microglobulin were used as housekeeping genes. Error bars show the standard deviation for n = 3 measurements from representative experiments.

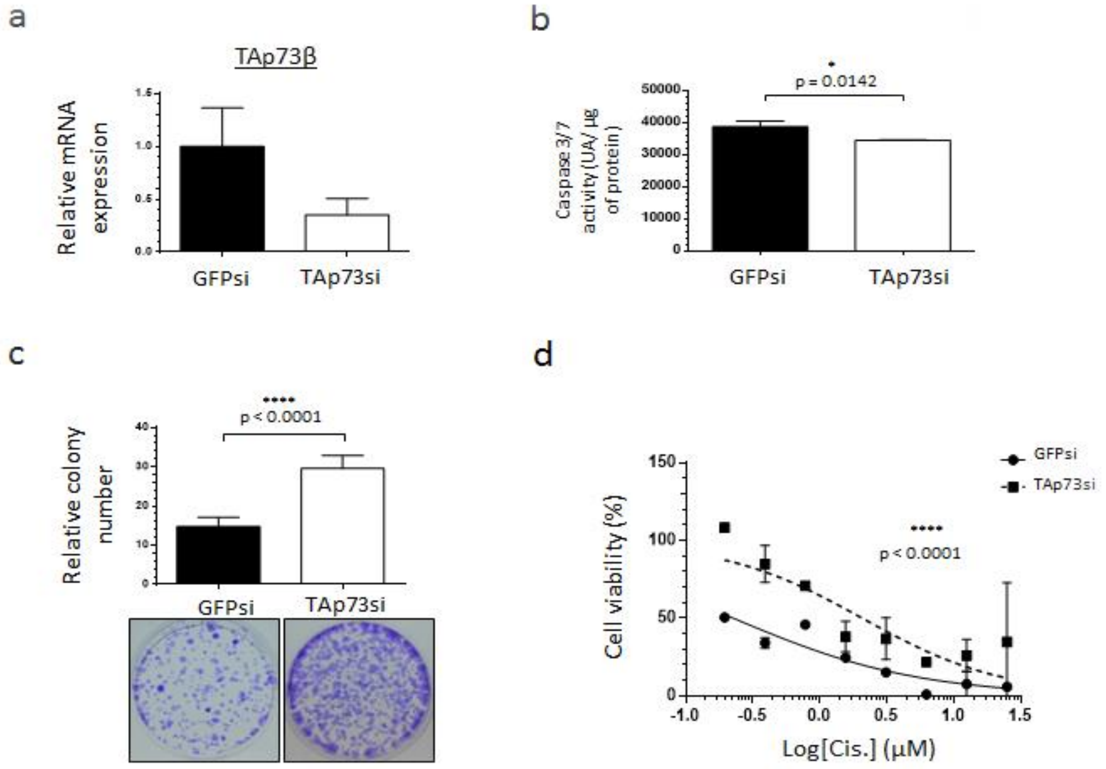

Supplementary Figure 3: TAp73β-mediated Cisplatin sensitivity of human Bone Sarcomas.

(a) The expression of TAp73β was evaluated by qRT–PCR in the 143B Osteosarcoma cell line, after infecting the cells with viral-supernatant of GFPsi- or TAp73si-transduced HEK 293 FT cells. Glyceraldehyde-3-phosphate dehydrogenase and β2-microglobulin were used as housekeeping genes for the qRT-PCR. (**b**) The basal apoptosis level was then evaluated by dosage of the caspase 3/7 activity in protein extracts from the same cells and in the same conditions as in (**a**). Error bars show the standard deviation for n = 3 measurements from representative experiments. (**c**) The basal clonogenic capabilities of the cells were evaluated in the same cells and in the same conditions as in (**a**). One thousand cells were seeded in 6-wells plates and incubated until the possibility of macroscopic clones counting. The cells were then fixed in glutaraldehyde and stained with Crystal Violet. Error bars show the standard

deviation for n = 3 measurements from representative experiments. Representative pictures of the wells in each condition were chosen. A two-tailed paired Student's *t-test* was used to compare the different conditions in the caspase 3/7 activity assays and in the clonogenic assays. (**d**) The same cells in the same conditions as in (**a**) were cultured for 48 h in the presence of Cisplatin at the indicated concentrations and cell viability was determined by WST-1 assay. The viability of the non-treated control of each cell line was assigned as 100%. A two-way ANOVA test was used to compare the different conditions in the viability assays.

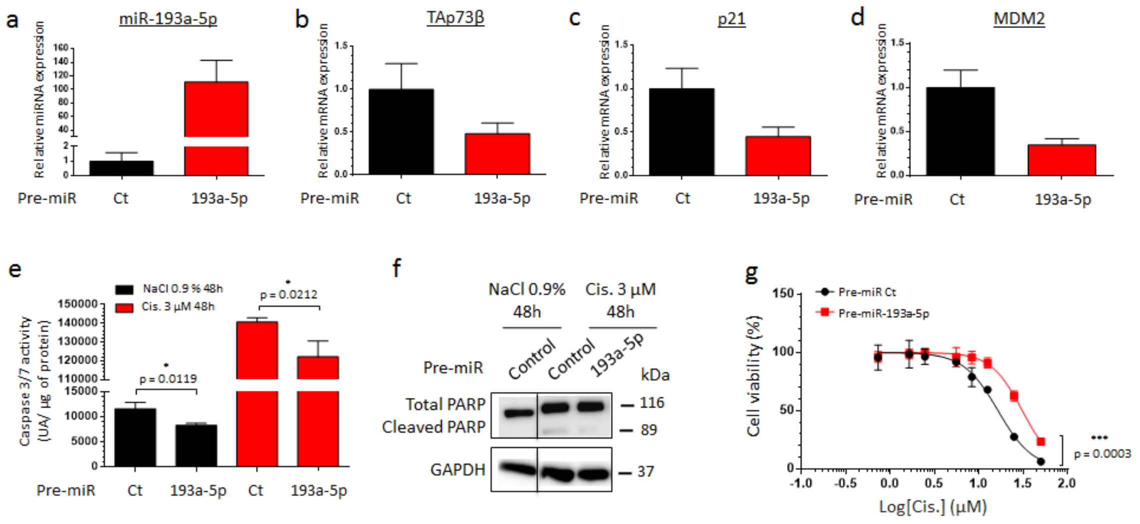

Supplementary Figure 4 | The TAp73β's targeting miR-193a-5p is implicated in the Cisplatin chemoresistance of human Bone Sarcomas.

(**a**) miR-193a-5p's expression was assessed by qRT–PCR in the SJSA-1 Osteosarcoma cell line forty-eight hours after either the pre-miR control or the pre-miR-193a-5p mimic's transfection. The expression of TAp73β (**b**), p21 (**c**) and MDM2 (**d**) were assessed by qRT–PCR in the same conditions as described in (**a**). RNU6B, Glyceraldehyde-3-phosphate dehydrogenase and β2-microglobulin were used as housekeeping genes for qRT-PCR and the error bars show the standard deviation for n = 3 measurements from representative experiments. (**e**) SJSA-1 Osteosarcoma cell line was transiently transfected in the same conditions as in (**a**) and was treated forty-eight hours later with 3 μM Cisplatin or the same amount of NaCl 0.9% for additional forty-eight hours. The apoptosis was then evaluated by dosage of the caspase 3/7 activity in protein extracts. Error bars show the standard deviation for n = 3 measurements from representative experiments. A two-tailed paired Student's *t-test* was used to compare the different conditions in the caspase 3/7 activity assays. (**f**) Protein's extracts from SJSA-1 Osteosarcoma cell line in same conditions as in (**a**) were subjected to Immunoblotting with anti-cleaved-PARP antibodies. Glyceraldehyde-3-phosphate

dehydrogenase was used as loading control. Data refer to three different experiments and western blot images are representative of these. Black lines show where the original gel was cropped to obtain the final image. **(g)** SJSA-1 Osteosarcoma cell line was transiently transfected with either pre-miR control or pre-miR-193a-5p mimics and was cultured forty-eight hours later in the presence of Cisplatin at the indicated concentrations for two hours. The cell viability was determined by WST-1 assay and compared with control. The viability of the non-treated control was assigned as 100%. A two-way ANOVA test was used to compare the different conditions in the viability assays.

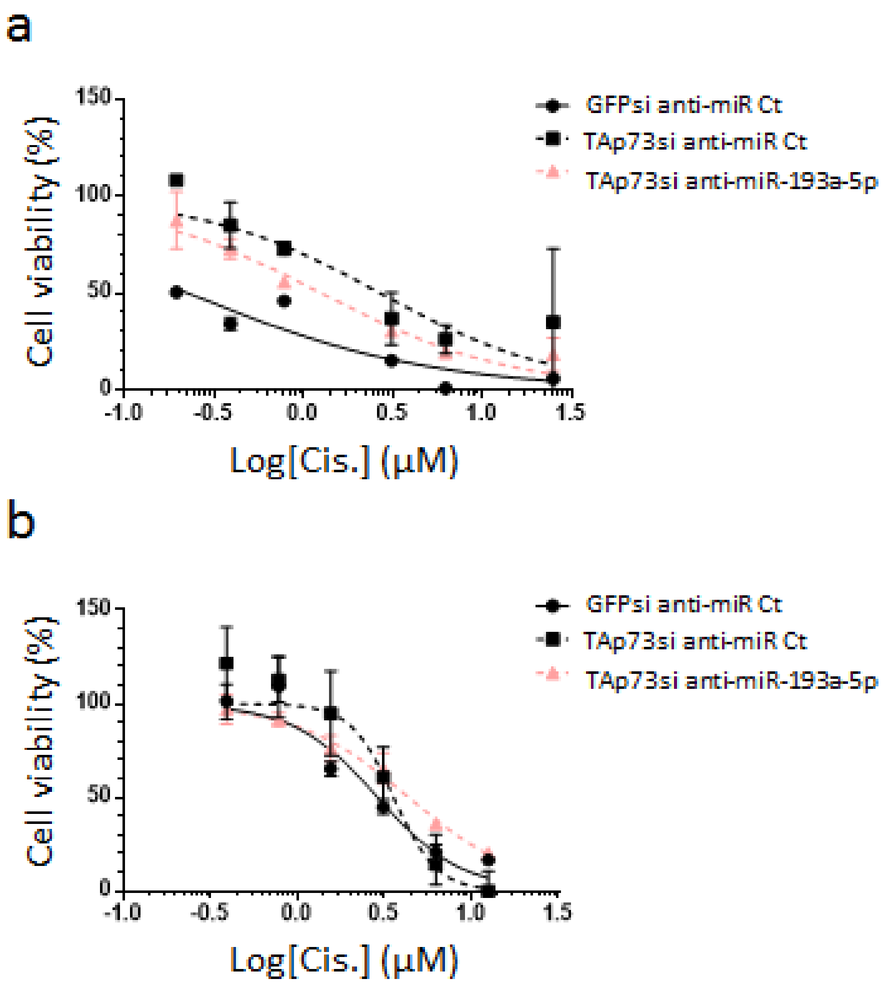

Supplementary Figure 5 | Inhibiting the miR-193a-5p increases the expression of TAp73β and restores the Cisplatin-sensitivity of human Bone Sarcoma cells.

The 143B Osteosarcoma cell line (**a**) and the RDES Ewing Sarcoma one (**b**) were infected with viral-supernatant of GFPsi -or TAp73si-transduced HEK 293 FT cells and stably express the GFP-siRNA or TAp73-siRNA. Those cell lines were then transfected with either the anti-

miR control or the anti-miR-193a-5p and were cultured forty-eight hours later in the presence of Cisplatin at the indicated concentrations for additional forty-eight hours. The cell viability was determined by WST-1 assay. The viability of the non-treated control of each cell line was assigned as 100%. A two-way ANOVA test was used to compare the different conditions in the viability assays
